# Topographically Localized Modulation of Tectal Cell Spatial Tuning by Complex Natural Scenes

**DOI:** 10.1523/ENEURO.0223-22.2022

**Published:** 2022-01-05

**Authors:** Thomas T. J. Sainsbury, Giovanni Diana, Martin P. Meyer

**Affiliations:** 1 The Centre for Developmental Neurobiology and MRC Center for Neurodevelopmental Disorders, King’s College London, London, United Kingdom, SE1 1UL; 2 Insitut Pasteur, University of Paris, Paris, France, 75015; 3 Sampled Analytics, Arcueil, France, 94110; 4 Lundbeck Foundation, Copenhagen, Denmark, 2100

**Keywords:** calcium imaging, contextual modulation, developmental biology, natural scenes, vision, zebrafish

## Abstract

The tuning properties of neurons in the visual system can be contextually modulated by the statistics of the area surrounding their receptive field (RF), particularly when the surround contains natural features. However, stimuli presented in specific egocentric locations may have greater behavioral relevance, raising the possibility that the extent of contextual modulation may vary with position in visual space. To explore this possibility, we utilized the small size and optical transparency of the larval zebrafish to describe the form and spatial arrangement of contextually modulated cells throughout an entire tectal hemisphere. We found that the spatial tuning of tectal neurons to a prey-like stimulus sharpens when the stimulus is presented against a background with the statistics of complex natural scenes, relative to a featureless background. These neurons are confined to a spatially restricted region of the tectum and have receptive fields centered within a region of visual space in which the presence of prey preferentially triggers hunting behavior. Our results suggest that contextual modulation of tectal neurons by complex backgrounds may facilitate prey-localization in cluttered visual environments.

## Visual Abstract

**Figure:**
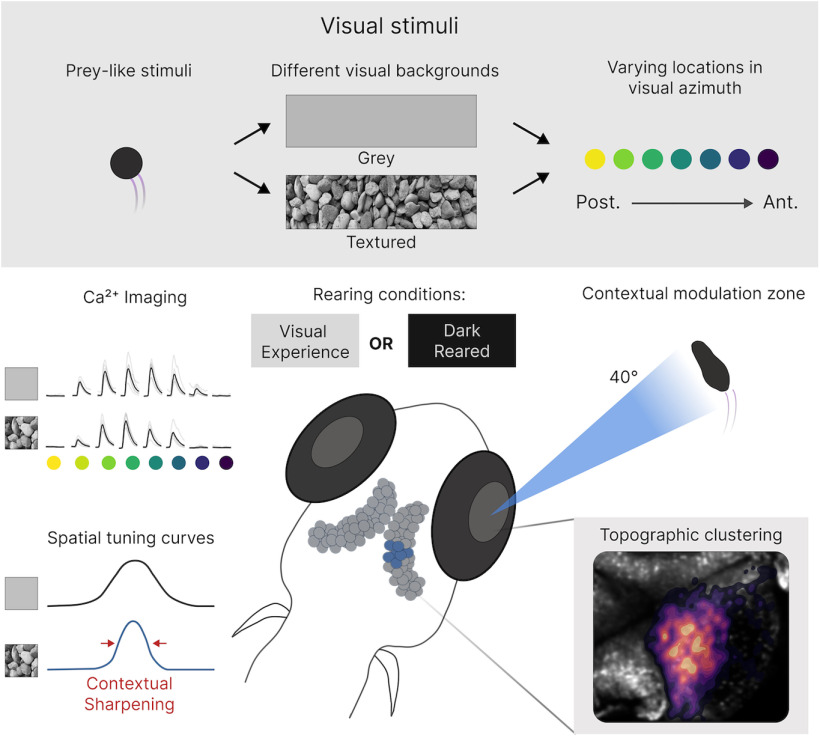

## Significance Statement

Visual neurons can have their responses contextually modulated by the statistics of the area surrounding the receptive field (RF). However, the spatial organization of these neurons throughout the visual system is virtually unknown. Here, we give the first demonstration that zebrafish tectal cells exhibit contextual modulation when stimuli are presented against a complex background. We find these contextually modulated cells are topographically restricted to a region visual space where hunting routines are preferentially triggered. Furthermore, we show that the circuits that support contextual modulation develop independently of visual experience (VE). These results suggest that the tectum contains hard-wired circuits that support contextual modulation and that this may improve the accuracy of prey localization at the onset of hunting routines in cluttered visual environments.

## Introduction

To survive in the wild, animals must be able to successfully detect and localize a food source in very different ecological niches. Even a single individual can experience visual environments that vary according to time of day, season and as they move from one place to another. How do visual systems enable successful food seeking behavior in dynamically changing visual environments? One potential neural mechanism that could be important is contextual modulation of circuit properties by the environment (Krause and Pack, 2014). Here, the firing properties of a neuron responding to a stimulus within its receptive field (RF) can be modulated by stimuli outside it (nRF; Allman et al., 1985; Levitt and Lund, 1997; Angelucci et al., 2002). Contextual modulation has be shown to affect tuning to size (Barlow, 1953; Hartline et al., 1956), contrast (Levitt and Lund, 1997), orientation (Okamoto et al., 2009), and for discriminating local motion (H.J. Sun et al., 2002; W. Sun et al., 2006). Furthermore, contextual modulation has been implicated in figure-ground separation (Allman et al., 1985), detexturization (Gheorghiu et al., 2014), generating “pop-out” phenomena (Knierim and van Essen, 1992; Zhaoping, 2008, 2016; Zhaoping and Zhe, 2012; Schmid and Victor, 2014; Ben-Tov et al., 2015) and sparsifying population activity that enhance coding efficiency (Vinje and Gallant, 2000, 2002; Haider et al., 2010). Importantly, recent studies have highlighted that these effects are most prominent when the nRF contains complex naturalistic spatial features and that the circuits that implement contextual modulation in mice require visual experience (VE) to develop (Guo et al., 2005; Pecka et al., 2014). Therefore, it has been suggested that contextual modulation in the visual system is important for detecting and localizing salient objects in complex natural environments and that the circuits that support contextual modulation are shaped by the statistics of natural scenes during development.

In the wild, zebrafish larvae inhabit environments that are often diverse and they hunt small mobile prey. In one instance an individual zebrafish may therefore hunt prey against a relatively featureless background such as the sky (as when larvae strike at the prey from below; Zimmermann et al., 2018; Mearns et al., 2020), while in another, they must strike at prey located in pebbles or plants (Engeszer et al., 2007). Hunting in zebrafish is driven by neural activity within the optic tectum which constitutes the largest visual area of the fish brain (Gahtan et al., 2005; Bianco and Engert, 2015; Romano et al., 2015; Avitan et al., 2020). In this study, we take advantage of the optical transparency and small size of the larval zebrafish brain to determine whether contextual modulation of tectal cell responses to prey-like stimuli by the visual scene could be a potential mechanism that supports hunting behavior in varied visual environments. We show that the spatial tuning of tectal neurons sharpens when the prey-like stimuli is presented against a complex visual background. Strikingly, we find that such modulation occurs within a spatially restricted tectal region. This region represents a point of visual space in which the presence of prey preferentially triggers hunting routines. Furthermore, we show that the circuits that support such contextual modulation do not require sensory experience for their proper development. Our results demonstrate that in larval zebrafish the contextual modulation of tectal neuron spatial tuning may facilitate prey localization in complex visual environments.

## Materials and Methods

### Animals and rearing

Calcium imaging experiments were conducted in transgenic zebrafish with pan-neuronal expression of nuclear-localized GCaMP6s *Tg(HuC:H2B-GCaMP6s; casper)* (Ahrens lab, Janelia farm). All larvae were raised at 28.5°C in Danieau solution (58 mm NaCl, 0.7 mm KCl, 0.4 mm MgSO_4_, 0.6 mm Ca(NO_3_)2, and 5 mm HEPES; pH 7.6) and were exposed to a 14/10 hr light/dark cycle. Zebrafish were raised in two rearing conditions. The first group was raised with visual experience (VE) in the form of small pieces of rock that were placed under the Petri dish, forming a bed of gravel. To assess the impact of visual experience on the contextual modulation in the tectum, the second rearing group were raised in total darkness (dark reared; DR). This ensured that they had no visual experience throughout development. Larvae from both groups were fed daily from 5 days post-fertilization (dpf) using live rotifiers. This work was approved by the local Animal Care and Use Committee and was conducted in accordance with the Animals (Experimental Procedures) Act, 1986, under license from the United Kingdom Home Office.

### Two-photon volumetric calcium imaging

Neural activity was monitored by imaging the calcium dynamics of between 500 and 1500 neurons in the tectal hemisphere that was contralateral to the eye recieving the visual stimulation with a custom built two-photon microscope (Independent NeuroScience Services). Excitation was provided by a Mai Tai HP ultrafast Ti:Sapphire laser (Spectraphysics) tuned to 940 nm. Laser power at the objective was kept below 18 mW for all fish. Emitted light was collected by a 16×, 1 NA water immersion objective (Nikon) and detected using a gallium arsenide phosphide detector (ThorLabs). Images (256 × 256 pixels) were acquired at a frame rate of 60 Hz by scanning the laser in the *x*-axis with a resonant scanner and in the *y*-axis by a galvo-mirror. The focal plane was adjusted in 15 μm steps using a piezo lens holder (Physik Instrumente). This allowed for volumetric data consisting of five focal planes to be collected at a volume rate of 9.7 Hz. Scanning and image acquisition were controlled by Scanimage Software (Vidrio Technologies). Each fish was imaged for 1 h.

### The visual stimulation setup

**Figure 1.**
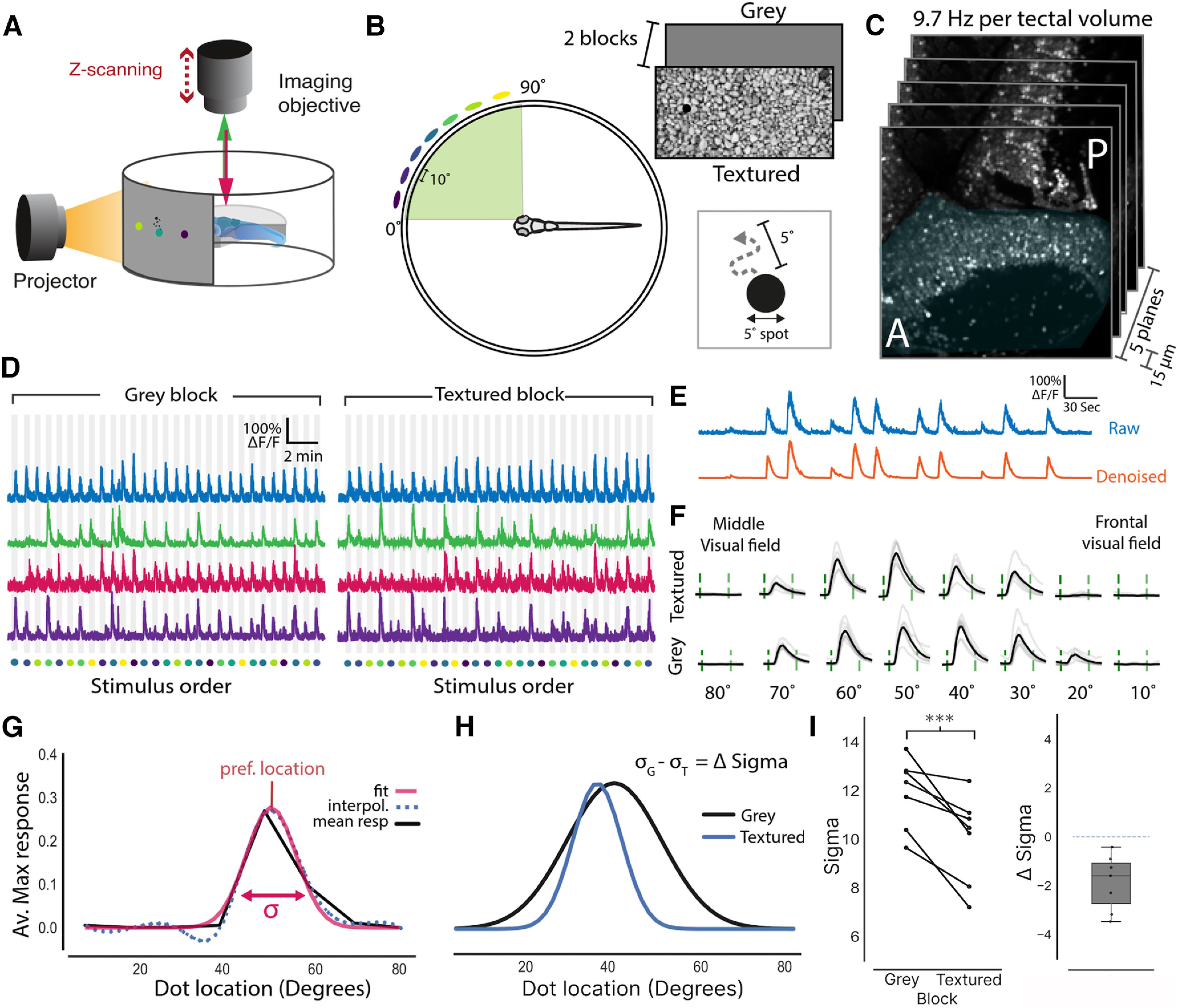
Presenting stimuli over a textured background sharpens the spatial tuning of tectal neurons. ***A***, Schematic of the imaging setup where larvae were head fixed in agarose allowing for visual stimuli to be projected onto a semicylindrical screen while neural activity is monitored via two-photon volumetric imaging. ***B***, A schematic showing that 5° moving dots (virtual prey) were presented at seven different locations along visual azimuth separated by 10°. These dots moved randomly within a 5° neighbored and were presented in two blocks with different backgrounds, gray screen or textured (gravel). A more extensive comparison between the statistics of these two backgrounds is provided in Extended Data Fig. 1-1). ***C***, Imaging volumes of the contralateral tectum (shaded blue) consisted of five optical sections that were taken 15 μm apart at an imaging speed of 9.7 Hz per volume. ***D***, Normalized fluorescence traces from cells that are active in both the gray and textured blocks. Stimulus location is color coded as in ***B***. ***E***, Raw fluorescence traces were denoised to generate smoothed calcium signals. ***F***, Mean responses to each of the stimulus locations (black) and individual repetitions (gray) for an example cell. Stimulus epoch start and end are indicated by the green dashed lines. ***G***, Spatial tuning curves for each cell were calculated by fitting a Gaussian to the interpolated average maximum response to each stimulus location. The preferred stimulus location was taken as the peak and tuning sharpness was taken as the standard deviation (σ) of the Gaussian fit. ***H***, Tuning fits for an example neuron for both the textured and gray backgrounds. From this, a neuron’s change in σ (Δ σ) can be calculated by subtracting its σ value for the gray background (*σ_G_*) from its σ for the textured background (*σ_T_*). ***I***, Left, Mean σ values for tectal neurons in both textured and gray blocks. Each connected line represents one fish (*n* = 7). Sigma was reduced for all fish in the textured block relative to the gray block (paired *t* test, *p* < 0.001). Right, A box plot showing the mean change in σ for each fish between the two blocks. Dotted line indicates zero change. This contextual modulation effect was found to be robust to the interpolation method that was used before fitting the Gaussian (Extended Data Fig. 1-2). To ensure that visual responses were not saturated in the these experiments a contrast sensitivity experiment was performed (Extended Data Fig. 1-3); ***p < 0.001.

To record visually evoked responses within the tectum, 7-dpf zebrafish were mounted in 2% agarose within a custom built perspex cylindrical chamber (Fig. 1*A*). The fish was positioned so that its right eye faced a semicircular screen covered in a gray diffusive filter and the chamber was filled with Danieau solution. This screen occupied 153° × 97° of visual azimuth and elevation, respectively, and was positioned 20 mm away from the fish. Visual stimuli could then be projected onto this screen using a P2JR pico-projector (AAXA Tech). To avoid interference of the projected image with the signal collected by the detector, a red long-pass filter (Zeiss LP590 filter) was placed in front of the projector.

10.1523/ENEURO.0223-22.2022.f1-1 Extended Data Figure 1-1 Stimulus background properties. ***A***, Images of the textured and grey backgrounds. Colored dots indicate the central locations of each of the prey-like stimuli, which are presented at these locations one at a time with five repeats within an imaging session. ***B***, The distribution of luminance in the anterior half of the textured background relative to the prey-like stimulus % luminance). While the backgrounds were not matched in mean luminance the backgrounds did display very similar mean luminance [grey background (grey dashed line) = 50%, textured background (blue dashed line) = 51%]. ***C***, Power spectral density maps for both backgrounds. ***D***, Averaged power spectral density *x* and *y* averaged over the textured background shows scale invariance, a feature that is typical of natural scenes (Van der Schaaf and Van Hateren, 1996). ***E–G***, To assess any local changes in luminance or spatial frequencies at each of the stimulus locations, 40° bounding boxes around each of the stimulus locations were calculated. Colors represent the position of the dots as in ***A***, ***B***. ***E***, Plot showing the mean and percentiles of the distribution of luminance around each of the prey-like stimulus locations. ***F***, Cumulative frequency density plots of the luminance around each of the stimulus locations was found to be similar for all stimulus locations [*p* > 0.05, Kolmogorov–Smirnov tests (KS-tests), Bonferroni corrected]. ***G***, Power spectral density plots for the area surrounding each of the stimulus locations was found to be similar for all locations (*p* > 0.05, KS-tests, Bonferroni corrected). Download Figure 1-1, TIF file.

**Figure 2.**
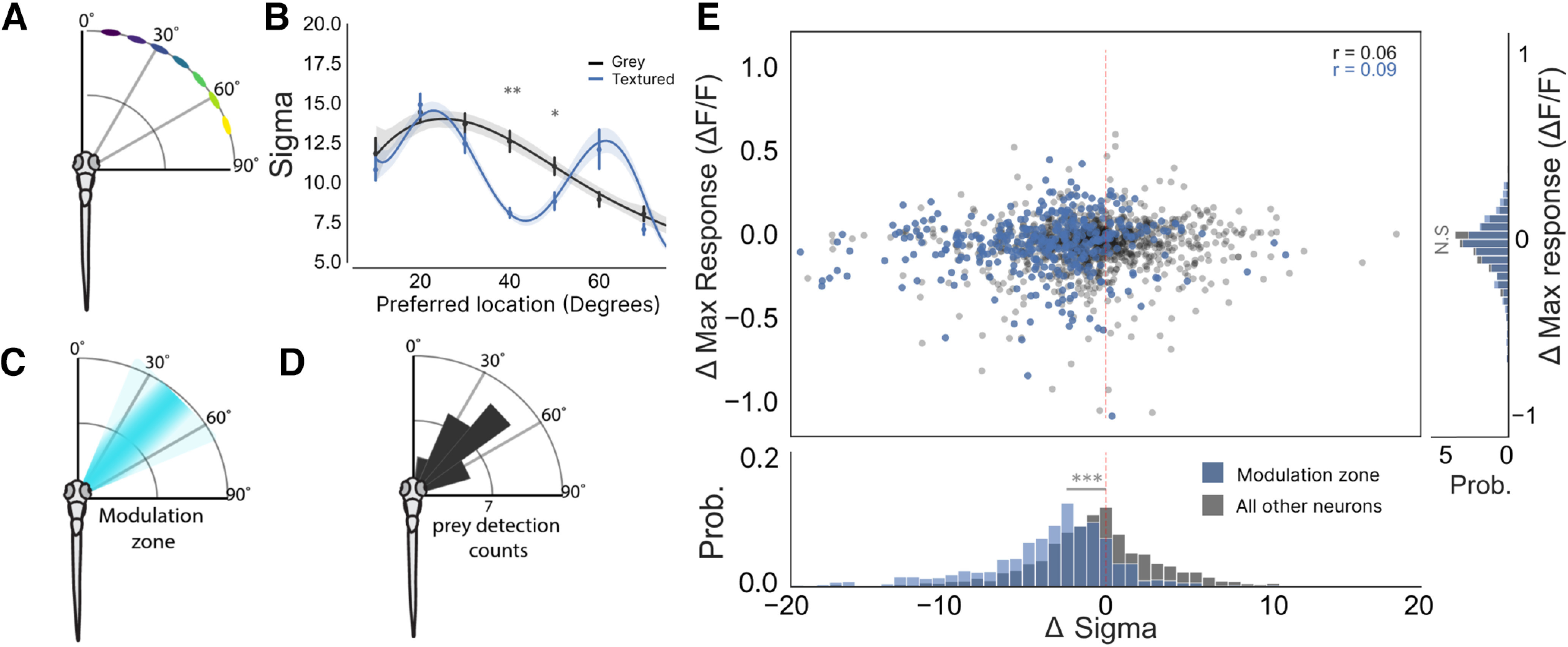
Contextual modulation takes place in a spatially restricted region of visual azimuth. ***A***, A schematic of stimulus location relative to the fish’s body axis. ***B***, A plot of σ against neuron’s preferred stimulus location for each fish, which demonstrates that spatial tuning exhibits contextual sharpening only for stimuli presented between 35° and 50° of visual azimuth (40°: *p* = 0.02, 50°: *p* = 0.05, two-way ANOVA, *t* tests with Bonferroni correction). ***C***, A schematic showing the zone in visual space where contextual modulation occurs. ***D***, This modulation zone corresponds to the area in visual space where hunting routines are preferentially triggered (modified from Romano et al., 2015). ***E***, Top, A scatter plot showing that a neuron’s change in maximum response is not correlated with its change in its σ [modulation zone neurons (blue) Pearson’s *r* = 0.09; all other tectal cells (dark gray) Pearson’s *r* = 0.06]. Cells within the modulation zone are highlighted in blue and were defined as cells which had a preferred tuning between 35° and 48°. Right, Histogram showing the difference in Δ max response for neurons within the modulation zone and all other tuned neurons (*p* = 0.7, *t* test). Bottom, Histogram showing the difference in Δσ for neurons within the modulation zone and all other tuned neurons (*p* < 0.001, *t* test); *p < 0.05, **p < 0.01, ***p < 0.001, N.S = not significant (*p* > 0.05).

10.1523/ENEURO.0223-22.2022.f1-2 Extended Data Figure 1-2 Contextual modulation effect in the tectum is robust to interpolation method. To ensure the tectal contextual modulation effect was not an artifact of the interpolation method this effect was reproduced using noninterpolated data. ***A***, Paired plot showing the change in the mean sigma of the spatial tuning curves when prey-like stimuli are presented against a grey or textured background. Each line represents one fish (*n* = 7). ***B***, A plot of sigma against neuron’s preferred stimulus location for each fish; *p < 0.05, **p > 0.01. Download Figure 1-2, TIF file.

**Figure 3.**
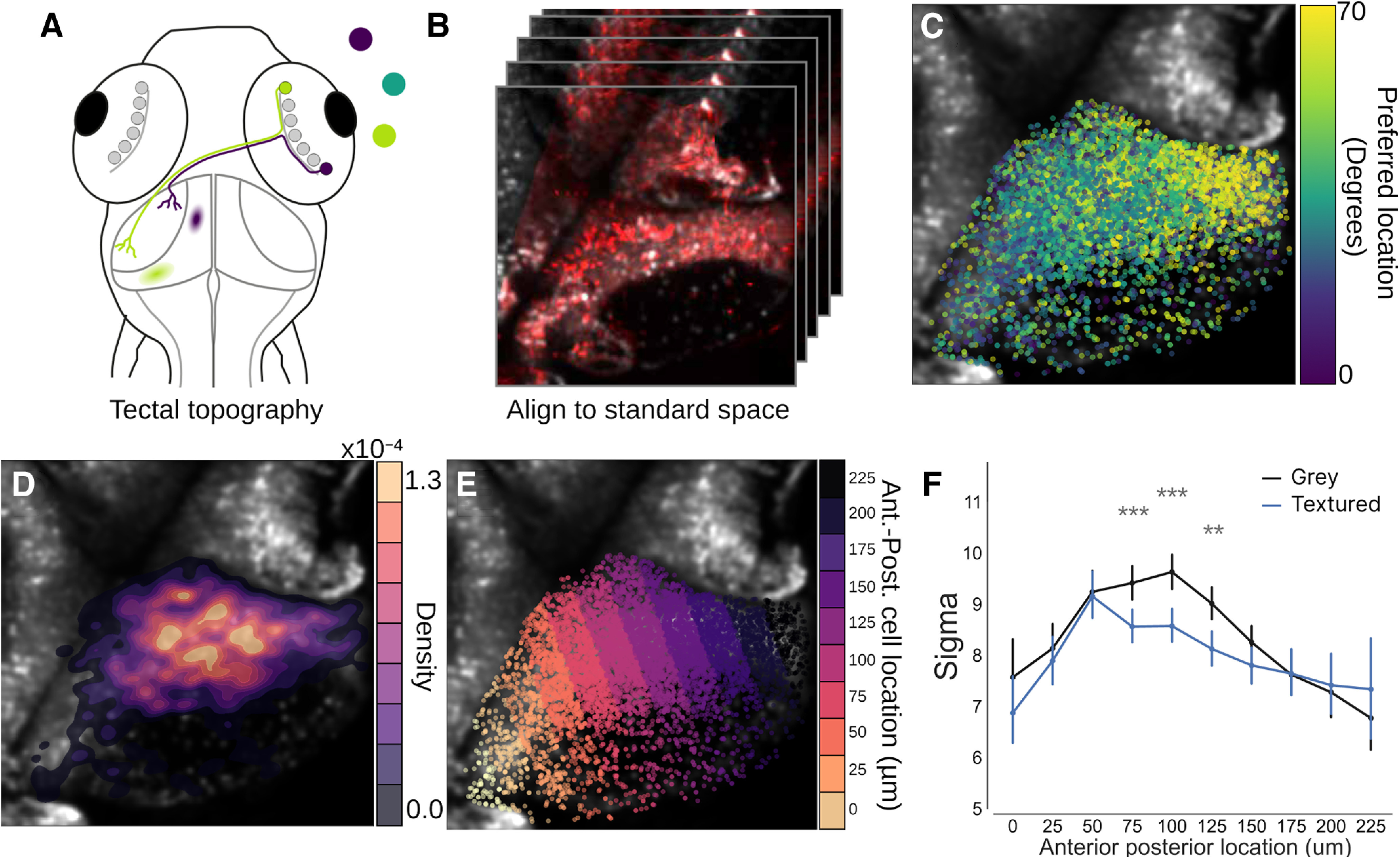
Modulated neurons are topographically distinct within the tectum. ***A***, Schematic detailing the topographic organization of the tectum. Here, retinal ganglion cells project out of the retina and make synapses in the neuropil of the contralateral hemisphere. They do this is a way that preserves a spatial map of visual space within the tectum with frontal visual space mapping onto the anterior portion of the tectum (purple), whereas rear visual space maps more posteriorly (lime green). ***B***, To assess the spatial arrangement of contextually modulated cells in the tectum a standard coordinate space was generated by aligning the functional imaging data to a high resolution stack of the tectum. ***C***, Overlay of cells in the tectum which have been colored by their tuning preference to demonstrate the topography of the tectum. ***D***, Overlay of a density heatmap showing the position of highly contextually modulated cells (Δ σ < −5) within the tectum. ***E***, To quantify the position of contextually modulated cells, the tectum was divided into bins along its anterior-posterior axis. ***F***, A plot of σ values for each segment within the anterior-posterior axis for both textured and gray backgrounds; **p < 0.01, ***p < 0.001.

10.1523/ENEURO.0223-22.2022.f1-3 Extended Data Figure 1-3 Contrast sensitivity of tectal neurons. To ensure that tectal responses were not saturating a contrast sensitivity experiment was performed where a single prey-like stimulus was presented to the fish at varying contrasts on a logarithmic scale from 5% to 100% contrast. The mean of the max responses in the tectum of each fish (*n* = 5) was plotted for each of the different contrasts. The dotted grey line represents the contrast between the grey background and the prey-like stimulus used in the contextual modulation experiment. Solid grey lines represent individual fish. Download Figure 1-3, TIF file.

### Visual stimuli

Visual stimuli were generated using a custom C^++^ script written by Giovanni Diana, Meyer lab. 5° black spots were presented at seven different locations in visual azimuth separated by 10° intervals (10°, 20°, 30°, 40°, 50°, 60°, 70°). 0° was defined as midline directly in front of the fish. These spots moved with motion that resembled rotifer movement within a neighborhood (5° radius) at a speed of 30°/s (Movie 1).

Movie 1. A movie showing the prey-like stimulus presented at seven different locations over the gray and textured backgrounds. Note: each stimulus in this movie is only presented once and the interstimulus intervals have been shortened to 2 s to shorten viewing time. 10.1523/ENEURO.0223-22.2022.video.1

These dots were presented in two blocks which differed in the background against which they were projected (Fig. 1*B*; Extended Data Fig. 1-1*A*). In one block the background was a digital photograph of the gravel that the fish were raised on (textured block) and the other it was simply a gray screen (gray block). In these blocks each spot was presented a total of seven times per block in 5-s epochs, followed by a 30-s interstimulus interval where only the background was presented. Importantly the movement of the dot was identical in each presentation. Both the order of the blocks and order of these spots within the blocks were pseudo-randomized. To prevent startling the fish, all dots faded in and out over the course of 1 s to minimize any startle effects that may be caused by sudden changes in the stimulus.

### Statistics of the gray and gravel backgrounds

In order to better understand how the visual properties of the two visual backgrounds may differ, both global and local statistics regarding the luminance and spatial frequencies were calculated. Global statistics were computed on the entire portion of visual space where the prey-like stimuli were presented (the anterior field of view; Extended Data Fig. 1-1*A*). The two backgrounds showed similar mean luminance (gray background = 50%, textured background = 51%; Extended Data Fig. 1-1*B*). These luminance values represent a percentage of the maximum dynamic range that could be displayed on the screen (with 256 being the maximum in an eight-bit image). Since the prey-like stimulus was presented with 0% luminance these values can also be interpreted as the contrast between the prey-like stimulus and the background. To compute the spatial frequencies that were contained within the background images, we took the Fourier transform for both the *x* and *y* dimensions and then calculated its power (Extended Data Fig. 1-1*C*). Although the gray background, which is uniform in its luminance, showed no distribution in its spatial frequencies, the textured background showed an extensive range of difference spatial frequencies. Averaging together the power-spectrum from the *x* and *y* dimensions indicated that this distribution was invariant across different spatial scales (Extended Data Fig. 1-1*D*). This is shown by the linear decay of the log power across different spatial frequencies. Such spatial invarience has been shown to be typical of naturalistic scenes such as forests and underwater scenes (Tolhurst et al., 1992; Van der Schaaf and Van Hateren, 1996; Balboa and Grzywacz, 2003; Roberts et al., 2022) and not is not present in more artificial stimuli such as urban and indoor environments (Flitcroft et al., 2020).

In addition to these global statistics, it is also important to ensure that the local statistics of the textured background surrounding each stimulus location do not significantly differ in their properties because such differences could lead to artifactual effects. To compute these local statistics, bounding boxes extending 20° either side of the center of the prey-like stimulus’s moving neighborhood were isolated. The distribution of luminance and spatial frequencies could then be compared between these regions. The mean luminance of these regions was similar to the gray background but did show a slight drift in mean luminace from anterior-posterior (10°: 49%, 20°: 49%, 30°: 48%, 40°: 47%, 50°: 47%, 60°: 46%, 70°: 46%; Extended Data Fig. 1-1*E*). However, this was still only a 4% difference in luminance from the gray background at the most posterior location. Furthermore, when comparing the full distributions of luminance, we found no significant differences between the stimulus locations (Extended Data Fig. 1-1*F*). The distribution of spatial frequencies at each of these stimulus locations was also found to be similar across the visual scene (Extended Data Fig. 1-1*G*). In summary, the main difference between these two backgrounds was in the varied distribution of luminance and spatial frequencies present in the textured background. Furthermore, distribution of luminance and spatial frequencies did not change significantly between the prey-like stimulus locations.

### Preprocessing of calcium imaging data

Visually evoked functional imaging data were both aligned and segmented using the Suite2p Python package (Pachitariu et al., 2016). Only segments within the tectum with a probability >0.5 of being a cell were used for further analysis.

To get smooth ΔF/F signals, free from imaging noise, the calcium signal was estimated from the raw fluorescence trace using the AR1 model contained within the OASIS package, with all parameters of the model being estimated from the data (Friedrich et al., 2017). These ΔF/F signals were then used to calculate tuning profiles for each cell.

### Generating tuning profiles

To generate tuning profiles the maximum response to the stimulus was calculated for each repetition by first taking the maximum amplitude for each stimulus presentation. These amplitudes were then averaged for each stimulus location across repetitions. These coarse grained responses were then interpolated with a cubic spline at 5° intervals to estimate the amplitude of responses between the presented stimulus (Avitan et al., 2020). These interpolated curves were then fitted with a Gaussian function with a baseline offset using nonlinear least squares. Initial parameters for fitting used the mean as the stimulus location eliciting response peak amplitude, and the initial value for SD was varied from low to high values. The highest goodness of fit (adjusted *r*^2^) was selected as the tuning profile for each neuron. This procedure was repeated twice for each cell, once for the gray block and once for the textured block. Only neurons with a goodness of fit greater that 0.9 in both blocks were used for further analysis. For these cells the mean of the Gaussian defined the preferred location, whereas its SD (σ) quantifies the sharpness of tuning. By taking the difference in σ (Δ σ) for each neuron between the textured and gray blocks the change in the neurons tuning could be quantified. These values could then be visualized against each neuron’s preferred location to understand the distribution of contextually modulated cells in visual space.

### Calculating the topographic arrangement of contextually modulated tectal neurons

To visualize topographic arrangement of contextually modulated cells in the tectum, cells from different fish needed to be transformed into a standard coordinate space. This required that the mean image for each functional imaging slice was aligned a reference two-photon stack of the entire tectum hemisphere. In this reference stack the *x* and *y* coordinates were the same but 200 slices in *z* were taken at a resolution of 2 μm. Functional imaging data were aligned to this reference stack using the “SyN” method contained in the ANTsPy package. This method performs nonrigid alignment by applying both affine and deformable transformations and uses the mutual information between both stacks of images as an optimization metric. The transformations from this alignment where the applied to the center point for each cell segment obtained using suite2p. This resulted in all neurons being put into a standardized coordinate space which could then be used to identify the position of contextually modulated tectal cells across fish.

Once in this space, principal component analysis was applied to the *x-y* positions of the tectal neuron segments. The first principle component spanned the major axis of the tectum, corresponding to the anterior-posterior axis. This axis could then be divided into bins and the σ values for each bin could be obtained.

### Contrast sensitivity analysis

To ensure that tectal responses were not saturated in our contextual modulation experiments in either of the backgrounds, a contrast sensitivity experiment was performed. In this experiment, a prey-like stimulus was presented to the fish in a single location. The contrast of the prey-like stimulus was then varied from 5% to 100% with five repeats at each contrast level. These presentations were presented in a pseudorandom order. Calcium imaging recordings were obtained and preprocessed using the same methods as the contextual modulation experiments. We then produced contrast sensitivity curves for cells that were responsive to the prey like stimulus by selecting cells that responded in at least four of the stimulus repeats to at least one of the contrast levels. The mean maximum response was then calculated across these repeats and across neurons. This produced a contrast sensitivity curve for each fish which could then be plotted together. These curves showed that visual responses to the stimulus could be seen from 26% contrast and saturated when the contrast exceeded 71% (Extended Data Fig. 1-3). Both backgrounds that were used for the contextual modulation experiment had contrasts that fell well within these bounds (gray: 50%, textured: 51%). This suggested that tectal responses would not be saturated when prey-like stimuli were viewed against either background.

### Sample sizes and statistical analysis

Multiple comparisons were first tested with and either one or two-way ANOVA depending on the number of factors being compared, unless otherwise stated. If significance was reached *post hoc t* tests with multiple comparisons were used with the method of correction. All tests and significance are reported in the figure legends throughout. All sample sizes are similar to those typically used in the zebrafish imaging field. All central points and error bars represent mean and SEM throughout.

## Results

Zebrafish have been seen to hunt prey from below (Zimmermann et al., 2018; Mearns et al., 2020), meaning that the prey would be seen against the relatively featureless background of the sky. However, a recent study of zebrafish habitats revealed that zebrafish are found abundantly in shallow rocky pools (Engeszer et al., 2007). Within these pools there would be circumstances where larvae would hunt prey against visual backgrounds displaying a range of contrasts and spatial frequencies. To determine whether neural responses in the zebrafish tectum are modulated according to the context of the visual scene, the optic tectum of 7-d postfertilization (dpf) larvae were imaged using two-photon volumetric imaging while stimuli were presented to the contralateral (right) eye. The stimuli consisted of prey-like stimuli (moving 5° black dots), that were displayed in a pseudorandom order at seven different locations along visual azimuth. These stimuli were displayed in two blocks that differed in their backgrounds. In one block the background contained naturalistic features in the form of a picture of gravel (textured block) whereas the other was a gray screen (gray block). The gray background was uniform in its luminance and therefore featureless. The textured background however was far more complex containing a range of different spatial frequencies and contrasts (Extended Data Fig. 1-1; see Materials and Methods for a full description). Within these two visual blocks the moving spot was presented seven times at each of the locations along the visual azimuth. We then examined how tuning to stimulus location (spatial tuning) was modulated by context (i.e., the background in each block; Fig. 1*A–E*).

Spatial tuning curves were fitted by taking the neuron’s mean response to each stimulus location and were interpolated with a cubic spline (Avitan et al., 2020). Then spatial tuning curves for each neuron were calculated by fitting this interpolated signal with a Gaussian (Fig. 1*F*,*G*). Tuning width, defined as the SD of the Gaussian fit (σ), could then be compared between blocks. This revealed that for all imaged fish (*n* = 7) the average tuning width was reduced when stimuli were viewed in the textured block relative to the gray block (Fig. 1*H*). Furthermore, the mean change in σ (Δ σ) for all fish was negative, with a mean reduction in σ of −1.8. Therefore, this suggests that the spatial tuning of tectal neurons to prey-like stimuli is sharpened when viewed within complex and naturalistic visual scenes. To ensure that this result was not an artifact of our interpolation fitting method, this same result was reproduced using noninterpolated data (Extended Data Fig. 1-2*A*), indicating that this effect is robust to the fitting procedure.

To examine whether contextual modulation occurs uniformly along visual azimuth, σ values for neurons in both blocks were plotted against their preferred stimulus location. This revealed that neurons with a preferred tuning location of 35–50° demonstrated significantly reduced σ values (Fig. 2*B*; see Extended Data Fig. 1-2*B* for noninterpolated data). Importantly, this area in visual space is where small orientating movements toward prey, known as “J-turns,” are most likely to be triggered (Fig. 2*C*,*D*; Romano et al., 2015; Lagogiannis et al., 2020).

One possible explanation for this sharpening of tuning could be that tectal responses to the prey-like stimuli are simply suppressed because of a reduction in contrast between the prey-like stimuli and the textured background at certain locations in visual azimuth. This could result in a reduction in the σ of the tuning curve. However, analysis of the properties of the two backgrounds showed that they were very similar in their mean luminance (gray background = 50%, textured background = 51%), suggesting that the mean contrast between the prey-like stimulus and the textured background is unlikely to drive a change in σ (Extended Data Fig. 1-1*B*; see Materials and Methods for full analysis). Despite this, localized differences in contrast in the area surrounding each of the stimulus locations were seen in the textured background with more posterior locations showing a slightly reduced contrast between the prey-like stimulus. However, even at the most posterior location this reduction was still very small (4% reduction in contrast relative to the gray background). In addition, this linear decay of contrast from anterior to posterior would not explain the localized contextual modulation effect that we see around 40°. Furthermore, we found no correlation between each neuron’s change in maximum response (Δ Maximum response) and Δ σ (Fig. 2*E*). This revealed that tectal neurons could detect the prey-like stimulus once the contrast surpassed 26% and responses tended to saturate once the contrast exceeded 71%. This suggests in our contextual modulation experiments where the contrast was only 50% the responses of tectal neurons were unlikely to be saturated. Together these results suggest that the spatial structure of the textured background sharpens the spatial receptive fields of tectal neurons without altering the amplitude of the neurons’ response at their preferred spatial locations.

Our results demonstrating that contextual modulation takes place within a defined region of visual azimuth suggest that contextually modulated tectal neurons are localized to a topographically restricted region of the tectum since the tectum contains a retinotopic map of visual space (Goodhill and Xu, 2005; Fig. 3*A*). To map the tectal location of contextually modulated neurons, all imaged fish were transformed into a standard coordinate system (see Materials and Methods; Fig. 3*B*). As expected, color-coding these neurons according to their preferred stimulus location revealed an ordered topographic map of visual azimuth space along the anterior-posterior axis of the tectum (Fig. 3*C*). To understand how contextually modulated cells are distributed within the tectum, the location of highly modulated cells (cells with a Δ σ < −5) were visualized as a density map over the tectum (Fig. 3*D*). This revealed that these cells tended to be grouped in the middle of the tectum’s anterior-posterior axis with a slight posterior bias. To quantify this topography the tectum was divided into zones along this axis and the mean σ was calculated for each zone for each stimulus block. This showed that only neurons in the middle zones of the tectum showed reduced σ values in the textured block. This effect was also visible when the mean Δ σ was calculated for each segment (Fig. 3*F*), showing that the center of the tectum showed large negative changes in σ that were not present at the tectal poles.

**Figure 4.**
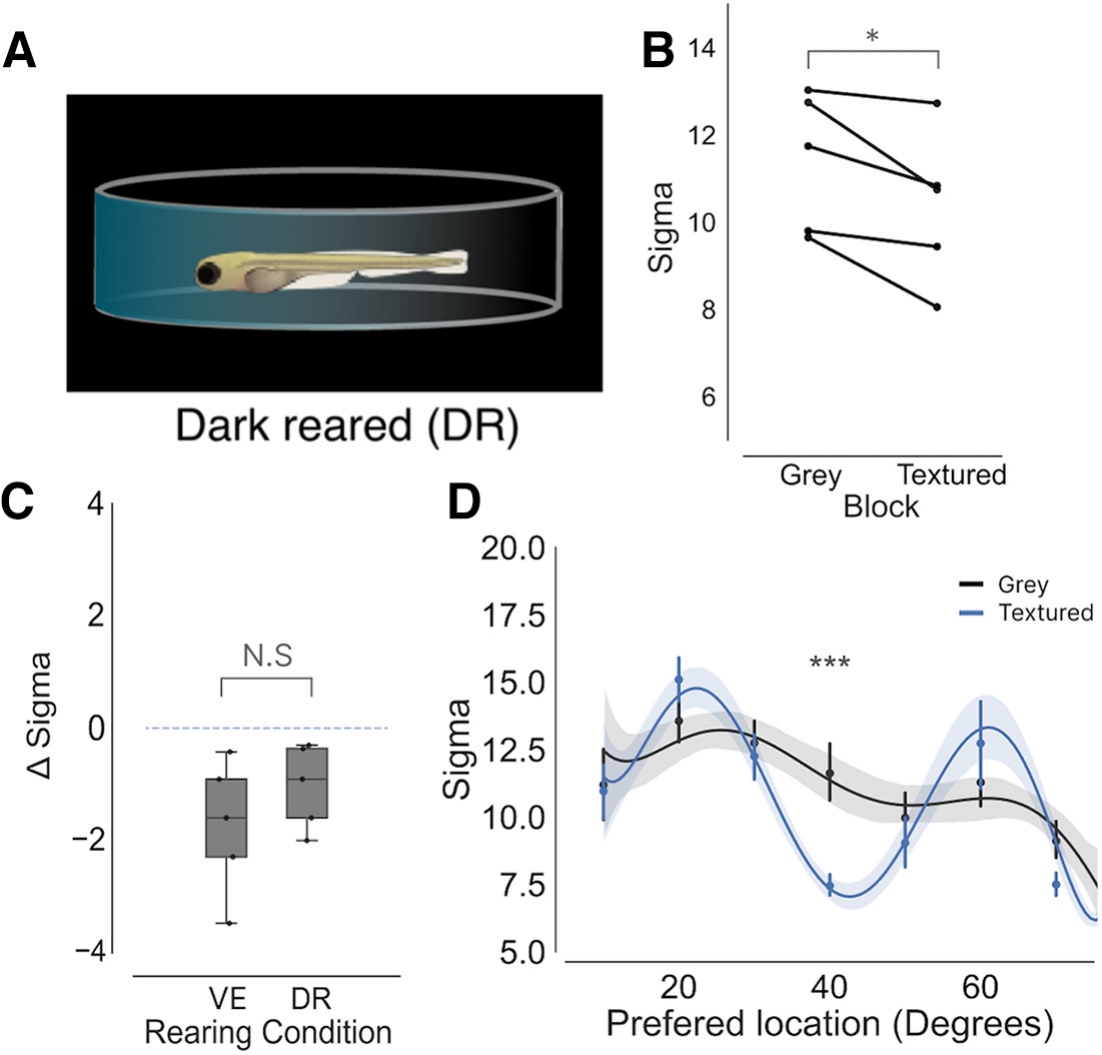
Visual experience has no effect on the development of contextual modulation in the optic tectum. ***A***, To alter the visual environment zebrafish were raised in complete darkness (DR, *n* = 5). ***B***, Plot mean σ for each DR fish during the gray and textured blocks (*p* = 0.03, paired *t* test). ***C***, A boxplot showing Δ σ between background for VE and DR fish (*p* = 0.1, *t* test). ***D***, A plot of σ against neuron’s preferred stimulus location for each DR fish. A similar modulation zone to is present around 40° (*p* < 0.001, *t* tests, Bonferroni corrected), which is similar to fish raised with visual experience; *p < 0.05, ***p < 0.001, N.S = not significant (*p* > 0.05).

In the visual cortex of mice, certain types of contextual modulation require visual experience to develop (Pecka et al., 2014). To test whether this was also the case for zebrafish, larvae were reared from 0- to 7-dpf dark reared (DR) to deprive them of visual stimuli (Fig. 4*A*). These fish could then be compared with the fish that had been reared with visual experience (VE) to understand whether the development of contextual modulation in the tectum requires visual experience. For all DR fish, a reduction in the mean σ was found when stimuli are viewed against the textured background relative to the gray background (Fig. 4*B*). By calculating the mean Δ σ for each DR fish revealed that the extent of contextual modulation was similar to that of VE fish (Fig. 4*C*). To determine whether a modulation zone was also present in DR fish the preferred location for each neuron was plotted against the σ value for both the textured and gray blocks. This revealed reduced σ values at 40° in the textured block, suggesting that a modulation zone was present in the same region of visual space as VE fish. These results demonstrate that development of circuits that support contextual modulation of spatial receptive fields in the zebrafish tectum are not dependent on visual experience.

## Discussion

Across multiple species it is well established that stimulating both a neuron’s RF and nRF with naturalistic stimuli can increase the selectivity of neurons for particular visual features (Vinje and Gallant, 2000; Haider et al., 2010; Pecka et al., 2014). Likewise, in our study, we find a subset of tectal neurons become more sharply tuned to the position of prey-like stimuli when presented against a picture of gravel. Such contextual modulation may reduce the uncertainty of the position of local objects. While the circuit mechanisms that enable contextual modulation in the zebrafish tectum are unknown, work from other species, including teleosts, have shown that it is brought about by GABAergic neurons with receptive fields that overlap with those of the modulated tectal neurons (Pecka et al., 2014; Ben-Tov et al., 2015; Zhaoping, 2016).

Interestingly, contextual modulation in the tectum was found not to be uniform, occurring in only a subset of tectal neurons that were clustered together in a posterior region of the tectum. The spatial receptive fields of neurons in this zone corresponds to a region in visual space where the presence of prey preferentially triggers the onset of hunting routines, characterized by J-turns which orientate the larvae toward the prey (McElligott and O’Malley, 2005; Bianco et al., 2011; Romano et al., 2015; Lagogiannis et al., 2020). Contextual modulation of tectal spatial receptive fields may enhance the accuracy of these orienting turns and thereby support successful hunting routines in cluttered visual environments.

While contextual modulation in mouse V1 occurs when the background contains spatial frequencies that are present in natural scenes, this property is not present at eye opening. Instead, it requires experience of natural features to develop (Pecka et al., 2014). In contrast, we found that contextual modulation in the optic tectum of zebrafish develops normally in fish that had been deprived of sensory experience. Our finding that contextual modulation develops independently of visual experience is consistent with the idea that many of the functional properties of the zebrafish tectum do not require visual experience for their development (Niell and Smith, 2005; Nevin et al., 2010). This suggests that certain aspects of the zebrafish visual system have evolved to be genetically hardwired, potentially to facilitate the rapid development of visually guided behaviors, such as hunting.

## Synthesis

Reviewing Editor: David Schoppik, New York University Decisions are customarily a result of the Reviewing Editor and the peer reviewers coming together and discussing their recommendations until a consensus is reached. When revisions are invited, a fact-based synthesis statement explaining their decision and outlining what is needed to prepare a revision will be listed below. The following reviewer(s) agreed to reveal their identity: Fumi Kubo.

The authors and I felt that the manuscript was quite clear and succinct in arguing that certain localized neurons in the zebrafish tectum show sharpener receptive fields when prey-like stimuli were presented on a textured background as compared to uniform grey. Below are a number of major and minor points for the authors to consider, should they wish to submit a revised manuscript

Major points:

1) The authors state that the spatial tuning of some tectal neurons to a prey-like stimulus sharpens in the presence of “the context of naturalistic visual scene (line 13, abstract)”. The title of the manuscript also emphasize “natural scenes”. However, it is not clear how naturalistic the visual stimulus they used for the calcium imaging. Please provide average size in visual angle of each grain contained in the gravel-textured background and discuss if this size is realistic. Also, the gravel background was presented to the side of the zebrafish. In natural habitats, gravel is most often located below the animals, not on the side. Please discuss how naturalistic the textured background used in the calcium imaging is in this context.

2) There is little explanation of the how the gravel background was designed and how it compared in properties to the uniform background. For example, was it matched in mean luminance? What was the overall contrast, and how did it compare to the stimulus? What was the spatial structure compared to the stimulus?

3) The authors argue that the dot contrast is lower in the gravel background case, but make this argument by quoting delta pixel values. Pixel values are not meaningful units here, and should be transformed to luminance values when necessary. Further, we are not sure average difference in pixel value is the best measure to judge how easy the stimulus is to detect. Perhaps, for example, the authors could compare the power spectrum of the stimulus and background.

4) It is argued that the sharpening of tuning could be due to an improvement in contrast on the gravel background, but you could also make the argument that a reduction of contrast could also sharpen tuning if the response is saturated at the peak. The authors make some arguments that this is not the case, but a simple and more direct experiment could be actually to vary the contrast of the stimulus on the uniform background and show that the same modulation in tuning is not observed.

5) Did the background stimulus vary from trial to trial or was it presented in a fixed position? This is important to interpret the dependence of contextual modulation on spatial location, but is not described.

6) Related to Point 1 above, it is not clear how similar the gravel-textured background used for the calcium imaging and the real gravel environment used in Figure 4. If they are not the same, can the authors exclude a possibility that gravel-reared fish did not receive a “proper” or “matching” visual experience, hence there was no change in the sharpening of tectal cells’ receptive fields?

7) In Figure 4C, to see the effect of rearing conditions on the change in tuning sharpeness, the authors should compare the gravel-reared (GR) and dark-reared (DR) conditions with normal-reared (NR) condition shown in Fig.1I (i.e. compare GR vs NR, DR vs NR). In the DR condition shown in Fig. 4B, it appears that the modulation zone (blue line) is located at around 35 degrees of the visual azimuth, whereas that of the normal reared condition is observed at just around 40 degrees (Fig. 2B). Is this possible shift statistically significant?

8) It is unclear why the authors interpolated the measurement curves before making the gaussian fits. Does this have a benefit over fitting directly to the measured data?

9) The last experiment testing the effect of rearing experience is based on a n of 4. As such, it is not clear how it could show a statistically significant difference between conditions, and we don’t really learn anything from the negative result.

Minor points:

1) It would be helpful if the authors can present the movement of prey-like stimulus (currently shown in Fig 1B) in a supplementary movie.

2) Figure 1I. The label of the last panel of Figure 1 should be “I”, but not “J”.

3) Figure 2C. What is plotted as the light blue shading? Is it a plot of real data or a schematic? Please clarify.

4) Figure 4B. Labels for the blue and black lines and a y-axis label are missing.

5) Please correct possible typos: Lines 25, 75, 167, 169, 173, 246, 257.

## Author Response

Response to the reviewers:

Synthesis Statement for Author (Required):

The authors and I felt that the manuscript was quite clear and succinct in arguing that certain localized neurons in the zebrafish tectum show sharpener receptive fields when prey-like stimuli were presented on a textured background as compared to uniform grey. Below are a number of major and minor points for the authors to consider, should they wish to submit a revised manuscript.

We thank the reviewers for their thoughtful comments. We believe that integrating these comments into the revised manuscript has helped to strengthen it. Below we detail the work we have done to address the reviewers’ comments. To aid the reviewer in identifying the changes that have been made we have provided a .pdf where additions have been highlighted in green.

Major points:

1) The authors state that the spatial tuning of some tectal neurons to a prey-like stimulus sharpens in the presence of “the context of naturalistic visual scene (line 13, abstract)”. The title of the manuscript also emphasize “natural scenes “. However, it is not clear how naturalistic the visual stimulus they used for the calcium imaging. Please provide average size in visual angle of each grain contained in the gravel-textured background and discuss if this size is realistic. Also, the gravel background was presented to the side of the zebrafish. In natural habitats, gravel is most often located below the animals, not on the side. Please discuss how naturalistic the textured background used in the calcium imaging is in this context.

We accept the reviewers’ point that zebrafish have been seen to hunt from below. When in open water, this would mean that they would be hunting against a relatively featureless background (the sky), similar to the grey background used in our study. However, zebrafish have been found in relatively shallow rocky pools which often have large banks surrounding them. Under some circumstances, particularly near the edges of these pools, zebrafish will hunt against cluttered backgrounds containing complex spatial frequencies such as pebbles. We therefore think it is an interesting question to ask if, and how, responses to prey-like stimuli vary according to the two different backgrounds. We acknowledge that we did not articulate this in the original version of the manuscript so we have done so in the revised version.

The textured background used in our study was a digital photograph of the pebble background that the fish were reared over. One feature that is typical of complex natural scenes such as forests or aquatic environments is that they exhibit spatial invariance in their spatial frequencies (Balboa and Grywacz 2003; Roberts et al., 2022). In the revised manuscript, we demonstrate that the textured background we use shows the same spatial invariance previously reported as a feature of complex natural scenes (Extended Data Fig 1-1C, D and H). This is shown by the linear decay when plotted on a log scale. We are unsure what constitutes a ‘realistic’ pebble size - the absolute size of pebbles and rocks vary considerably in the real world, as does the retinal image’s size according to distance.

We will address points 2 and 3 together since they are related:

2) There is little explanation of the how the gravel background was designed and how it compared in properties to the uniform background. For example, was it matched in mean luminance? What was the overall contrast, and how did it compare to the stimulus? What was the spatial structure compared to the stimulus?

3) The authors argue that the dot contrast is lower in the gravel background case, but make this argument by quoting delta pixel values. Pixel values are not meaningful units here, and should be transformed to luminance values when necessary. Further, we are not sure average difference in pixel value is the best measure to judge how easy the stimulus is to detect. Perhaps, for example, the authors could compare the power spectrum of the stimulus and background.

We agree with the reviewers that a more detailed analysis of the statistical properties of the gravel background is needed. Therefore we have created a new Extended Data Figure comparing the two backgrounds (Extended Data Figure 1-1) alongside a detailed explanation of the analysis of these two backgrounds (see “Statistics of the grey and textured backgrounds” section of the materials and methods, Page 3). We should note that in doing this analysis we found that we were loading the incorrect image of the textured background (a screenshot of the curved projected image which has similar naming), which resulted in the wrong delta pixel values being quoted in the original manuscript. We have updated this and, as requested, quote luminance values in the revised manuscript. These luminance values are a percentage of the maximum dynamic range that could be displayed on the screen (with 256 being the maximum in 8-bit image). Such percentages are reproducible across different systems and dynamic ranges. Since the luminance of the prey-like stimulus was 0% luminance, these luminance values also represent the contrast between the background and the stimulus.

While we did not match the mean luminance between the grey background and the textured background, we found that the mean luminance was similar between these two backgrounds (grey background - 50% luminance, textured background - 51%, Extended Data Figure 1-1B). In addition to mean luminance, we also calculated localised luminance within bounding boxes around each stimulus location (Extended Data Figure 1-1 E-F). For all prey-like stimulus locations in the textured background, we found a slight reduction in contrast between the background and prey-like stimulus relative to the grey background (10º: -1%, 20º: -1%, 30º: -2% 40º: -3%, 50º: -3%, 60º: -4%, 70º: -4%). While we do observe a slight gradient from anterior to posterior, the difference between them is still very small (<5%). In addition to comparing mean luminance values, we also compared the full distributions of luminance around each stimulus location and found no significant differences between them, suggesting that local differences in luminance would not be a contributing factor to the localised contextual modulation we see around 40º.

To assess differences in the spatial structure between the backgrounds, we took the average power spectrum of the x and y dimensions of the two images. The grey background, which is uniform in its luminance, showed no detectable spatial frequencies in its power (Extended Data Fig 1C). The textured background however displayed an extensive range of spatial frequencies, which showed linear decay on a log scale (Extended Data Figure 1-11D). This suggests that the textured background shows a distribution of spatial frequencies typically found within complex natural scenes. Furthermore, we found no difference in the distribution of local spatial frequencies in the areas surrounding each of the stimulus locations, indicating that these spatial frequencies were found uniformly across the anterior portion of the visual scene (Extended Data Figure 1-1G).

4) It is argued that the sharpening of tuning could be due to an improvement in contrast on the gravel background, but you could also make the argument that a reduction of contrast could also sharpen tuning if the response is saturated at the peak. The authors make some arguments that this is not the case, but a simple and more direct experiment could be actually to vary the contrast of the stimulus on the uniform background and show that the same modulation in tuning is not observed.

We agree that if responses were saturated in the grey background condition, a reduction in contrast in the textured background could result in no change in the maximum response, whilst still giving a change in sigma. However, varying the contrast of a stimulus at multiple locations with the same number of repeats over the two backgrounds would have led to an unfeasibly long imaging time.

Instead, we performed a simpler experiment to understand the point at which visual responses to the prey-like stimulus in the tectum saturated. In this experiment, a prey-like stimulus was presented over a uniform background and the contrast of the dot was varied from 5-100% on a logarithmic scale (Extended Data Figure 1-3, and the experimental design is outlined in the “Contrast sensitivity analysis” section of the Materials and Methods, Page 5). We find that tectal neurons start to respond to the stimulus at approximately 26% and begin to saturate when the contrast exceeds 76%. Therefore, in our contrast sensitivity experiments, where the grey and textured background had a mean contrast of 50% and 51%, the neural responses were unlikely to be saturated.

5) Did the background stimulus vary from trial to trial or was it presented in a fixed position? This is important to interpret the dependence of contextual modulation on spatial location, but is not described.

This is an important point because the fact that this contextual modulation effect occurs at a particular point in visual space could reflect a non-uniform distribution of the gravel background statistical properties. Whilst the textured background was presented in a fixed position, we find that the statistical properties of the textured background in the area surrounding each of the stimulus locations were similar.

To demonstrate this, we took bounding boxes surrounding each of the stimulus locations to calculate the localised statistical properties of the textured background. Calculating the distribution of luminance and power spectrum of spatial frequencies within each of these boxes did not reveal any differences in the statistical properties of the background across the visual scene. This indicates that local differences in the background are unlikely to be a factor in driving the non-uniformity of contextual modulation in tectal neurons. These findings are detailed in a new Extended Data Figure 1-1E-G).

Here we shall address points 6, 7 and 9 together since they are related in their explanation:

6) Related to Point 1 above, it is not clear how similar the gravel-textured background used for the calcium imaging and the real gravel environment used in Figure 4. If they are not the same, can the authors exclude a possibility that gravel-reared fish did not receive a “proper” or “matching” visual experience, hence there was no change in the sharpening of tectal cells’ receptive fields?

7) In Figure 4C, to see the effect of rearing conditions on the change in tuning sharpness, the authors should compare the gravel-reared (GR) and dark-reared (DR) conditions with normal-reared (NR) condition shown in Fig.1I (i.e. compare GR vs NR, DR vs NR). In the DR condition shown in Fig. 4B, it appears that the modulation zone (blue line) is located at around 35 degrees of the visual azimuth, whereas that of the normal reared condition is observed at just around 40 degrees (Fig. 2B). Is this possible shift statistically significant?

9) The last experiment testing the effect of rearing experience is based on a n of 4. As such, it is not clear how it could show a statistically significant difference between conditions, and we don’t really learn anything from the negative result.

It seems that we did not make this clear within the text but we only had 2 rearing conditions throughout the paper:

1) Those fish that had visual experience (GR - in the original manuscript). - We have changed the name of this group to be the visual experience group (VE) for clarity in the revised manuscript. The gravel background used in the contextual modulation experiments was a digital photo of the gravel that these fish were raised on to ensure that these fish received properly matched visual experience.

2) Those fish that have no visual experience, through dark rearing (DR).

In the original paper, we pooled together data from VE and DR fish throughout figures 1-3 and then separated them out into their rearing conditions to see if contextually modulated cells develop in the absence of visual experience. We realise that this was confusing so therefore updated how we did this analysis. Now figures 1-3 include only VE fish and we only include the DR fish in figure 4. In addition, we have added 1 VE fish and 1 DR fish to increase the n of these experiments.

The point of figure 4 was not to test if there is an effect in the VE fish (since this was essentially the baseline group) but to instead ask the question: “Do contextually modulated cells develop in the absence of visual experience?” To answer this we examined the effect of dark rearing. The reason this is an interesting question to ask is that in contextual modulation within the visual cortex of mice does not develop when they are dark reared (Pecka et al., 2014). This suggests that visual experience is required to shape cortical circuits that are necessary for contextual modulation. Therefore in figure 4, we wanted to see if this was also true for zebrafish by looking at whether there is contextual modulation present within DR fish. We find that there is still a strong contextual modulation effect in these fish suggesting that the circuits that support contextual modulation do not require visual experience for their proper development. Therefore, we do not view this result as a negative result.

In regards to there being a possible shift in tuning between the VE and DR fish, adding the extra DR fish removed the appearance of this shift. In this dataset the modulation zone is centred at 40 degrees, which is similar to the VE fish. Therefore no further test is needed.

8) It is unclear why the authors interpolated the measurement curves before making the gaussian fits. Does this have a benefit over fitting directly to the measured data?

Gaussian fitting by linear least squares is a powerful method for estimating gaussian fits. However, it has been shown to be biassed by noise when sample sizes are small, leading to unreliable r-squared values. In order to limit imaging times in our study, we coarsely sampled the visual scene by displaying the prey-like stimulus at 7 different locations, with multiple repeats at each location. To ensure that our fits were not biassed by this coarse-graining we used the commonly used method of cubic spline interpolation to estimate responses between our sampled locations as done in Avitan et al., 2019. However, to ensure that our results are not an artefact of this interpolation we checked that the contextual modulation effect could be reproduced in non-interpolated data (Extended Data Figure 1-2). We find that tectal neurons on average sharpened their tuning profile when prey-like stimuli are presented against the textured background and that this effect occurs in neurons that are tuned to approximately 40 degrees. This shows that the results of the paper are robust to this interpolation method.

Minor points:

1) It would be helpful if the authors can present the movement of prey-like stimulus (currently shown in Fig 1B) in a supplementary movie.

We have added a movie of the stimulus (movie 1). Note: this movie only contains 1 repeat per stimulus location, and the interval between stimuli has been shortened so that all of the stimuli can be viewed within a shorter amount of time.

2) Figure 1I. The label of the last panel of Figure 1 should be “I”, but not “J”.

We have updated the figure.

3) Figure 2C. What is plotted as the light blue shading? Is it a plot of real data or a schematic? Please clarify.

This is a schematic showing where the modulation zone is located relative to where fish typically turn towards prey. We have updated the figure legend to make this clear.

4) Figure 4B. Labels for the blue and black lines and a y-axis label are missing.

We have updated the figure.

5) Please correct possible typos: Lines 25, 75, 167, 169, 173, 246, 257.

All of these identified typos have been corrected.

References:

Avitan, L., Pujic, Z., Mölter, J., McCullough, M., Zhu, S., Sun, B., Myhre, A. E., and Goodhill, G. J. (2020). Behavioral Signatures of a Developing Neural Code. Current biology : CB, 30(17):3352-3363.

Balboa, R. M. and Grzywacz, N. M. (2003). Power spectra and distribution of contrasts of natural images from different habitats. Vision Research, 43(24):2527-2537.

Pecka, M., Han, Y., Sader, E., and Mrsic-Flogel, T. D. (2014). Experience-Dependent Specialization of Receptive Field Surround for Selective Coding of Natural Scenes. Neuron, 84(2):457-469

Roberts, M. M., Schira, M. M., Spehar, B., and Isherwood, Z. J. (2022). Nature in motion: The tuning of the visual system to the spatiotemporal properties of natural scenes. Journal of Vision, 22(6):7-8.

## References

[B1] Allman J, Miezin F, McGuinness E (1985) Stimulus specific responses from beyond the classical receptive field: neurophysiological mechanisms for local-global comparisons in visual neurons. Annu Rev Neurosci 8:407–430. 10.1146/annurev.ne.08.030185.002203 3885829

[B2] Angelucci A, Levitt JB, Walton EJ, Hupe JM, Bullier J, Lund JS (2002) Circuits for local and global signal integration in primary visual cortex. J Neurosci 22:8633–8646. 10.1523/JNEUROSCI.22-19-08633.2002 12351737 PMC6757772

[B3] Avitan L, Pujic Z, Mölter J, McCullough M, Zhu S, Sun B, Myhre AE, Goodhill GJ (2020) Behavioral signatures of a developing neural code. Curr Biol 30:3352–3363.e5. 10.1016/j.cub.2020.06.040 32710821

[B4] Balboa RM, Grzywacz NM (2003) Power spectra and distribution of contrasts of natural images from different habitats. Vision Res 43:2527–2537. 10.1016/s0042-6989(03)00471-1 13129540

[B5] Barlow HB (1953) Summation and inhibition in the frog’s retina. J Physiol 119:69–88. 10.1113/jphysiol.1953.sp004829 13035718 PMC1393035

[B6] Ben-Tov M, Donchin O, Ben-Shahar O, Segev R (2015) Pop-out in visual search of moving targets in the archer fish. Nat Commun 6:6476. 10.1038/ncomms7476 25753807

[B7] Bianco IH, Engert F (2015) Visuomotor transformations underlying hunting behavior in zebrafish. Curr Biol 25:831–846. 10.1016/j.cub.2015.01.042 25754638 PMC4386024

[B8] Bianco IH, Kampff AR, Engert F (2011) Prey capture behavior evoked by simple visual stimuli in larval zebrafish. Front Syst Neurosci 5:101. 10.3389/fnsys.2011.00101 22203793 PMC3240898

[B9] Engeszer RE, Patterson LB, Rao AA, Parichy DM (2007) Zebrafish in the wild: a review of natural history and new notes from the field. Zebrafish 4:21–40. 10.1089/zeb.2006.9997 18041940

[B10] Flitcroft DI, Harb EN, Wildsoet CF (2020) The spatial frequency content of urban and indoor environments as a potential risk factor for myopia development. Invest Ophthalmol Vis Sci 61:42. 10.1167/iovs.61.11.42 PMC7533745 32986814

[B11] Friedrich J, Zhou P, Paninski L (2017) Fast online deconvolution of calcium imaging data. PLoS Comput Biol 13:e1005423. 10.1371/journal.pcbi.1005423 28291787 PMC5370160

[B12] Gahtan E, Tanger P, Baier H (2005) Visual prey capture in larval zebrafish is controlled by identified reticulospinal neurons downstream of the tectum. J Neurosci 25:9294–9303. 10.1523/JNEUROSCI.2678-05.2005 16207889 PMC6725764

[B13] Gheorghiu E, Kingdom FA, Petkov N (2014) Contextual modulation as de-texturizer. Vision Res 104:12–23. 10.1016/j.visres.2014.08.013 25204771

[B14] Goodhill GJ, Xu J (2005) The development of retinotectal maps: a review of models based on molecular gradients. Network 16:5–34. 10.1080/09548980500254654 16353341

[B15] Guo K, Robertson RG, Mahmoodi S, Young MP (2005) Centre-surround interactions in response to natural scene stimulation in the primary visual cortex. Eur J Neurosci 21:536–548. 10.1111/j.1460-9568.2005.03858.x 15673453

[B16] Haider B, Krause MR, Duque A, Yu Y, Touryan J, Mazer JA, McCormick DA (2010) Synaptic and network mechanisms of sparse and reliable visual cortical activity during nonclassical receptive field stimulation. Neuron 65:107–121. 10.1016/j.neuron.2009.12.005 20152117 PMC3110675

[B17] Hartline HK, Wagner HG, Ratliff F (1956) Inhibition in the eye of limulus. J Gen Physiol 39:651–673. 10.1085/jgp.39.5.651 13319654 PMC2147566

[B18] Knierim JJ, van Essen DC (1992) Neuronal responses to static texture patterns in area V1 of the alert macaque monkey. J Neurophysiol 67:961–980. 10.1152/jn.1992.67.4.961 1588394

[B19] Krause MR, Pack CC (2014) Contextual modulation and stimulus selectivity in extrastriate cortex. Vision Res 104:36–46. 10.1016/j.visres.2014.10.006 25449337

[B20] Lagogiannis K, Diana G, Meyer MP (2020) Learning steers the ontogeny of an efficient hunting sequence in zebrafish larvae. eLife 9:e55119. 32773042 10.7554/eLife.55119 PMC7561354

[B21] Levitt JB, Lund JS (1997) Contrast dependence of contextual effects in primate visual cortex. Nature 387:73–76. 10.1038/387073a0 9139823

[B22] McElligott MB, O’Malley DM (2005) Prey tracking by larval zebrafish: axial kinematics and visual control. Brain Behav Evol 66:177–196. 10.1159/000087158 16088102

[B23] Mearns DS, Donovan JC, Fernandes AM, Semmelhack JL, Baier H (2020) Deconstructing hunting behavior reveals a tightly coupled stimulus-response loop. Curr Biol 30:54–69.e9. 10.1016/j.cub.2019.11.022 31866365

[B24] Nevin LM, Robles E, Baier H, Scott EK (2010) Focusing on optic tectum circuitry through the lens of genetics. BMC Biol 8:126. 10.1186/1741-7007-8-126 20920150 PMC2949621

[B25] Niell CM, Smith SJ (2005) Functional imaging reveals rapid development of visual response properties in the zebrafish tectum. Neuron 45:941–951. 10.1016/j.neuron.2005.01.047 15797554

[B26] Okamoto M, Naito T, Sadakane O, Osaki H, Sato H (2009) Surround suppression sharpens orientation tuning in the cat primary visual cortex. Eur J Neurosci 29:1035–1046. 10.1111/j.1460-9568.2009.06645.x 19291228

[B27] Pachitariu M, Stringer C, Schröder S, Dipoppa M, Rossi LF, Carandini M, Harris KD (2016) Suite2p: beyond 10,000 neurons with standard two-photon microscopy. bioRxiv e061507.

[B28] Pecka M, Han Y, Sader E, Mrsic-Flogel TD (2014) Experience-dependent specialization of receptive field surround for selective coding of natural scenes. Neuron 84:457–469. 10.1016/j.neuron.2014.09.010 25263755 PMC4210638

[B29] Roberts MM, Schira MM, Spehar B, Isherwood ZJ (2022) Nature in motion: the tuning of the visual system to the spatiotemporal properties of natural scenes. J Vis 22:7–8. 10.1167/jov.22.6.7 35587355 PMC9123491

[B30] Romano SA, Pietri T, Pérez-Schuster V, Jouary A, Haudrechy M, Sumbre G (2015) Spontaneous neuronal network dynamics reveal circuit’s functional adaptations for behavior. Neuron 85:1070–1085. 10.1016/j.neuron.2015.01.027 25704948 PMC4353685

[B31] Schmid AM, Victor JD (2014) Possible functions of contextual modulations and receptive field nonlinearities: pop-out and texture segmentation. Vision Res 104:57–67. 10.1016/j.visres.2014.07.002 25064441 PMC4253048

[B32] Sun HJ, Zhao J, Southall TL, Xu B (2002) Contextual influences on the directional responses of tectal cells in pigeons. Vis Neurosci 19:133–144. 10.1017/s0952523802191127 12385626

[B33] Sun W, Deng Q, Levick WR, He S (2006) ON direction-selective ganglion cells in the mouse retina. J Physiol 576:197–202. 10.1113/jphysiol.2006.115857 16901944 PMC1995646

[B34] Tolhurst DJ, Tadmor Y, Chao T (1992) Amplitude spectra of natural images. Ophthalmic Physiol Opt 12:229–232. 10.1111/j.1475-1313.1992.tb00296.x 1408179

[B35] Van der Schaaf A, Van Hateren JH (1996) Modelling the power spectra of natural images: statistics and information. Vision Res 36:2759–2770. 10.1016/0042-6989(96)00002-8 8917763

[B36] Vinje WE, Gallant JL (2000) Sparse coding and decorrelation in primary visual cortex during natural vision. Science 287:1273–1276. 10.1126/science.287.5456.1273 10678835

[B37] Vinje WE, Gallant JL (2002) Natural stimulation of the nonclassical receptive field increases information transmission efficiency in V1. J Neurosci 22:2904–2915. 10.1523/JNEUROSCI.22-07-02904.2002 11923455 PMC6758304

[B38] Zhaoping L (2008) Attention capture by eye of origin singletons even without awareness - A Hallmark of a bottom-up saliency map in the primary visual cortex. J Vis 8:1. 10.1167/8.5.1 18842072

[B39] Zhaoping L (2016) From the optic tectum to the primary visual cortex: migration through evolution of the saliency map for exogenous attentional guidance This review comes from a themed issue on Systems neuroscience. Curr Opin Neurobiol 40:94–102. 10.1016/j.conb.2016.06.017 27420378

[B40] Zhaoping L, Zhe L (2012) Properties of V1 neurons tuned to conjunctions of visual features: application of the V1 saliency hypothesis to visual search behavior. PLoS One 7:e36223. 10.1371/journal.pone.0036223 22719829 PMC3373599

[B41] Zimmermann MJ, Nevala NE, Yoshimatsu T, Osorio D, Nilsson DE, Berens P, Baden T (2018) Zebrafish differentially process color across visual space to match natural scenes. Curr Biol 28:2018–2032.e5. 29937350 10.1016/j.cub.2018.04.075

