# Supplementary figures and images for: Topographically Localized Modulation of Tectal Cell Spatial Tuning by Complex Natural Scenes
Source: eNeuro. 2022 Jan 5;10(1):ENEURO.0223-22.2022. doi: 10.1523/ENEURO.0223-22.2022 (PMC9833049; doi:10.1523/ENEURO.0223-22.2022)

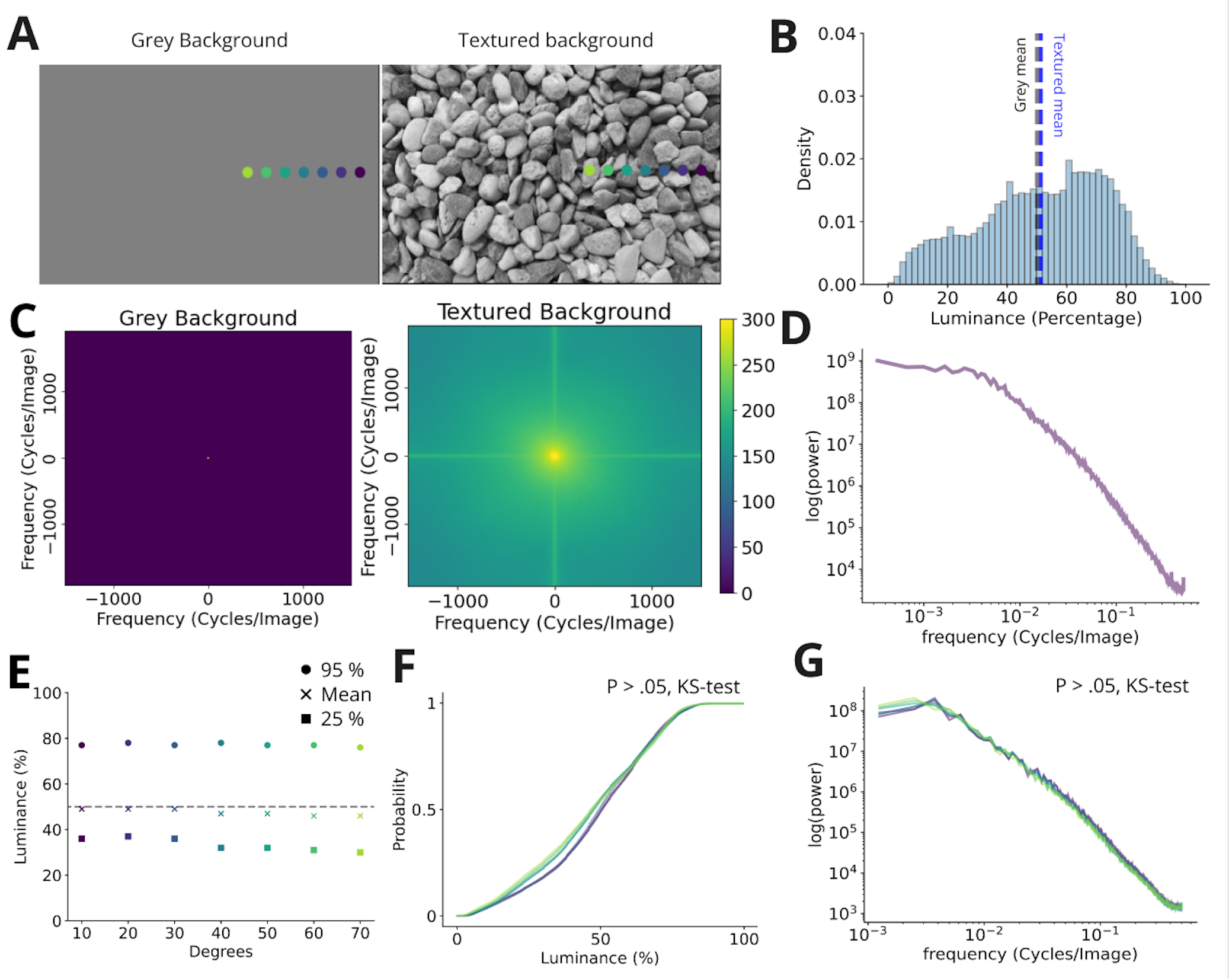

Supplement: Extended Data Figure 1-1 — Stimulus background properties. A, Images of the textured and grey backgrounds. Colored dots indicate the central locations of each of the prey-like stimuli, which are presented at these locations one at a time with five repeats within an imaging session. B, The distribution of luminance in the anterior half of the textured background relative to the prey-like stimulus % luminance). While the backgrounds were not matched in mean luminance the backgrounds did display very similar mean luminance [grey background (grey dashed line) = 50%, textured background (blue dashed line) = 51%]. C, Power spectral density maps for both backgrounds. D, Averaged power spectral density x and y averaged over the textured background shows scale invariance, a feature that is typical of natural scenes (Van der Schaaf and Van Hateren, 1996). E–G, To assess any local changes in luminance or spatial frequencies at each of the stimulus locations, 40° bounding boxes around each of the stimulus locations were calculated. Colors represent the position of the dots as in A, B. E, Plot showing the mean and percentiles of the distribution of luminance around each of the prey-like stimulus locations. F, Cumulative frequency density plots of the luminance around each of the stimulus locations was found to be similar for all stimulus locations [p > 0.05, Kolmogorov–Smirnov tests (KS-tests), Bonferroni corrected]. G, Power spectral density plots for the area surrounding each of the stimulus locations was found to be similar for all locations (p > 0.05, KS-tests, Bonferroni corrected). Download Figure 1-1, TIF file. [file enu-eN-NWR-0223-22-s07.tif]

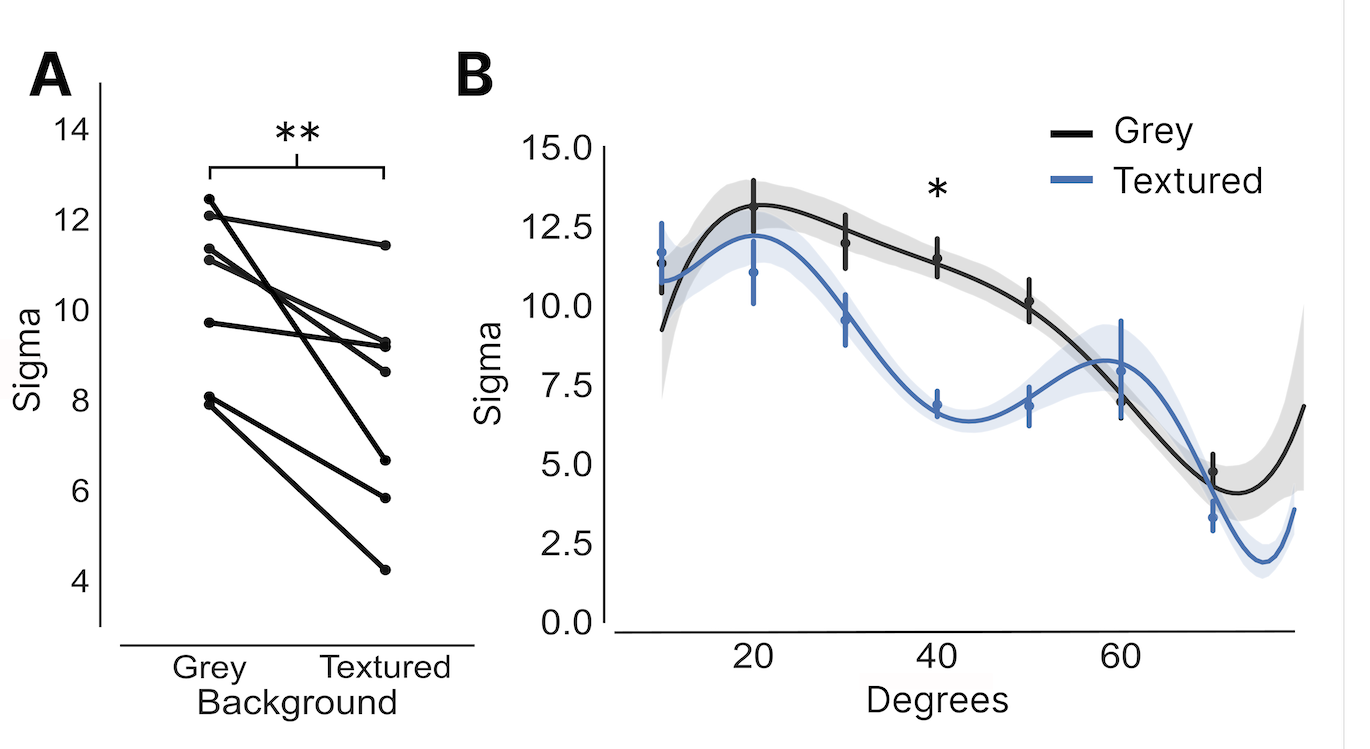

Supplement: Extended Data Figure 1-2 — Contextual modulation effect in the tectum is robust to interpolation method. To ensure the tectal contextual modulation effect was not an artifact of the interpolation method this effect was reproduced using noninterpolated data. A, Paired plot showing the change in the mean sigma of the spatial tuning curves when prey-like stimuli are presented against a grey or textured background. Each line represents one fish (n = 7). B, A plot of sigma against neuron’s preferred stimulus location for each fish; *p < 0.05, **p > 0.01. Download Figure 1-2, TIF file. [file enu-eN-NWR-0223-22-s08.tif]

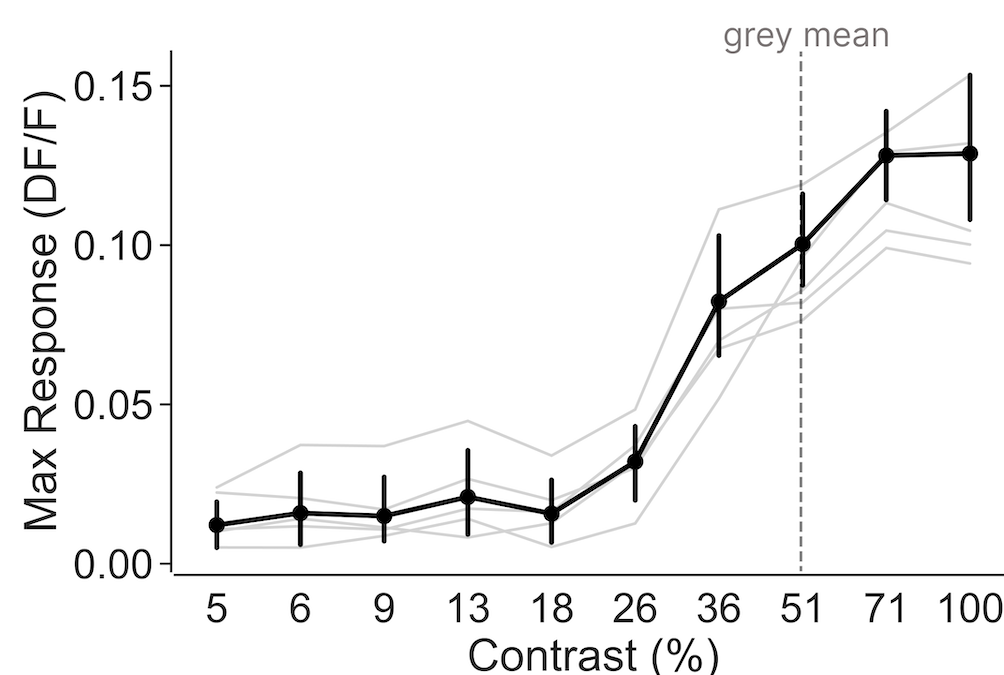

Supplement: Extended Data Figure 1-3 — Contrast sensitivity of tectal neurons. To ensure that tectal responses were not saturating a contrast sensitivity experiment was performed where a single prey-like stimulus was presented to the fish at varying contrasts on a logarithmic scale from 5% to 100% contrast. The mean of the max responses in the tectum of each fish (n = 5) was plotted for each of the different contrasts. The dotted grey line represents the contrast between the grey background and the prey-like stimulus used in the contextual modulation experiment. Solid grey lines represent individual fish. Download Figure 1-3, TIF file. [file enu-eN-NWR-0223-22-s09.tif]
